# Optimizing multi-supplier multi-item joint replenishment problem for non-instantaneous deteriorating items with quantity discounts

**DOI:** 10.1371/journal.pone.0246035

**Published:** 2021-02-08

**Authors:** Xueyi Ai, Yi Yue, Haoxuan Xu, Xudong Deng

**Affiliations:** 1 School of Management, Wuhan University of Science and Technology, Wuhan, China; 2 School of Business Administration, Zhongnan University of Economics and Law, Wuhan, China; University of Defence in Belgrade, SERBIA

## Abstract

This paper deals with a new joint replenishment problem, in which a number of non-instantaneous deteriorating items are replenished from several suppliers under different quantity discounts schemes. Involving both joint replenishment decisions and supplier selection decisions makes the problem to be NP-hard. In particular, the consideration of non-instantaneous deterioration makes it more challenging to handle. We first construct a mathematical model integrated with a supplier selection system and a joint replenishment program for non-instantaneous deteriorating items to formulate the problem. Then we develop a novel swarm intelligence optimization algorithm, the Improved Moth-flame Optimization (IMFO) algorithm, to solve the proposed model. The results of several numerical experiments analyses reveal that the IMFO algorithm is an effective algorithm for solving the proposed model in terms of solution quality and searching stableness. Finally, we conduct extensive experiments to further investigate the performance of the proposed model.

## 1. Introduction

In real life, the decay or deterioration of products is a natural phenomenon. The majority of sales revenue of supermarkets and grocery stores derives from deteriorating merchandises. For example, deteriorating items contribute 50% of retailing sales in North American and 30% of supermarket sales worldwide. (Chen et al. 2014 [1]). With the development of preservation technologies, retailers could guarantee products not to deteriorate immediately upon produced, resulting in a longer shelf life. This is now commonly applied for almost all food items (e.g., firsthand vegetables, milk, meats and sea foods), pharmaceuticals, fashionable products, and electronic equipment. Wu et al. (2006) [2] initially introduced the term of “non-instantaneous deterioration” to define the phenomenon, and developed an inventory replenishment model for non-instantaneous deteriorating items with stock-dependent demand and partial backlogging. Subsequently, a large number of studies have emerged to consider the inventory problems for non-instantaneous deteriorating items under various settings. However, seldom researchers consider the multi-item inventory policy for non-instantaneous deteriorating products in a multi-supplier setting, whereas it is a well common problem in practice. To fill this gap, this study takes a further step on the existing works to consider such a problem.

Among multi-item inventory models, the joint replenishment policy shows advantages of economic benefit in two respects (Khouja and Goyal, 2008 [3]). On the one hand, it pursues the savings on major ordering costs; on the other, it may enjoy the discounted order quantities. As a result, it has been applied by many practitioners. For example, in B2C E-Commerce operations, Jingdong and Amazon take considerable attentions on deciding the best way to group more items in one batch during the replenishment to share the fixed major ordering cost. Likewise, when dealing with products with deterioration, it is common that retailers replenish various fresh produce (e.g., fruits, firsthand vegetables, etc.) simultaneously. For instance, Zhongbai holdings Group Co. Ltd., a retailer in Wuhan City, Hubei Province, China, has built a huge warehouse where various kinds of fresh products are delivered jointly. The joint replenishment policy is also suitable under such settings.

However, when considering the perishability feature of products with deterioration, it is more realistic for retailers to seek multiple suppliers to replenish. For example, Walmart has recently built ten more warehouse centers for fresh products supplied from over 8500 more global suppliers. Although fresh products contribute almost 30% margin profit, they also bring a huge loss. Over years, Walmart has taken considerable attentions on the joint replenishment policy for these perishable items. Multiple suppliers usually offer different quantity discounts to stimulate the demand, and thus to increase the market share. Nevertheless, an extra purchasing could also impel to the extra loss of products. Therefore, in the multi-item joint replenishment problem with multiple suppliers, how many perishable items to be replenished altogether and which specific item should be replenished from which supplier become key but challenging decisions for retailers to minimize the total cost.

Motivated by such a realistic problem, this study firstly considers a multi-supplier joint replenishment problem for non-instantaneous deteriorating items (MS-JRNID) with several different quantity discounts offered by suppliers. As the joint replenishment problem (JRP) and the supplier selection problem are both NP-hard (Arkin et al. 1989 [4]; Moon et al. 2008 [5]), an additional consideration of non-instantaneous deterioration makes the problem more complicated, and hard to be solved by traditional algorithms of JRP. To tackle such an intricate problem, it is reasonable to apply population-based meta-heuristic algorithms, which show advantages of providing a set of non-dominated solutions in a single run and not suffering from the curse-of-dimensionality (Ganguly, 2020 [6]). As the math-flame optimization (MFO) algorithm is a novel swarm intelligence optimization algorithm, it has been extensively used in practice and turns out to be a competitor in the field of complicated optimization due to its high efficiency (Mirjalili. 2015 [7]). However, as a neoteric swarm intelligence optimization technology, the MFO algorithm suffers from premature convergence (Zhang et al., 2016 [8]). As a result, we develop an improved moth-flame optimization algorithm to solve our problem. The improvement mainly lies in that a hyperbolic spiral function is employed to improve the accuracy and speed of the algorithm, and the Levy-Flight function is added to avoid falling into a local optimum.

This study is an extension of our previous work (Ai et al. 2017 [9]) by involving multiple suppliers offering different quantity discounts. Since the main motivation for retailers to adopt the joint replenishment policy is to obtain the discounted order quantities from suppliers, such considerations make this research more practical and advantageous. Firstly, a multi-supplier multi-item joint replenishment model is formulated for non-instantaneous deteriorating items. Secondly, a novel IMFO algorithm is proposed to solve the model. Thirdly, numerical experiments are conducted to demonstrate the performance of the proposed algorithm and the effectiveness of the proposed model. Finally, parameter sensitivity analyses are investigated and managerial insights are derived.

The remainder of the paper is organized as follows. Section 2 reviews the related literature. In Section 3, assumptions, notations and the MS-JRNID model formulation are presented. Section 4 introduces the original MFO algorithm and the proposed IMFO algorithm. Numerical examples and parameter sensitive analyses are conducted in Section 5. Section 6 draws the conclusion and depicts the future research.

## 2. Literature review

Our study is of relevance to three research streams: inventory models of non-instantaneous deteriorating products, the research of the JRP model as well as its extensions and heuristics to solve these models, and the applications of the MFO algorithm.

1 Inventory models for non-instantaneous deteriorating items

Given the practical and theoretical relevance, inventory models of non-instantaneous deteriorating products have been extensively investigated since the work of Wu et al. (2006) [2]. As pioneer researchers, they proposed an inventory replenishment model for non-instantaneous deteriorating items with stock-dependent demand to determine the optimal replenishment policy. Thereafter, scholars have focused on inventory problems of the non-instantaneous deterioration items under varies conditions. Tat et al. (2015) [10] proposed an economic order quantity model for non-instantaneous deteriorating items with and without shortages to investigate the performance of a vendor-managed inventory system. Ghasemi (2015) [11] developed a classical economic production quantity model for non-instantaneous deteriorating items by considering a relationship between the holding cost and the ordering cycle length. Pal et al. (2018) [12] developed an inventory model for non-instantaneous deteriorating items with a preservation technology and a random staring time of deterioration. In their model, shortages are allowed or partially backlogged. Mishra et al. (2020) [13] developed a sustainable supply chain inventory policy with controllable non-instantaneous deterioration and environmental policy for greenhouse items.

All of the above studies focus on single-item problems, while only a few researchers pay attentions to multi-item inventory problems with non-instantaneous deterioration. For example, Li et al. (2007) [14] proposed EOQ-based models of perishable products to investigate the impact of the postponement strategy on retailers. Xu and Xiao (2013) [15] developed a JRP model for non-instantaneous deteriorating items with quantity discounts in a supply chain of one-supplier and one-retailer. With full backlogging allowed, Ai et al. (2017) [9] designed an optimal joint replenishment policy for multiple non-instantaneous deteriorating items under constant demand rate. Our research follows the stream by not only considering the inventory replenishment problem for multiple non-instantaneous deteriorating items, but also in a multi-supplier setting.

2 JRP models and algorithms

Over the last few decades, the JRP has been extensively investigated. Reviews on related works up to the late 1980s were conducted by Goyal and Satir (1989) [16], as well as Aksoy and Erenguc (1988) [17]. Later on, a comprehensive literature review of the JRP related studies from 1989 to 2005 is available in Khouja and Goyal (2008) [3]. Over the past few years, researchers have focused on the studies on the JRP and its extensions, in which the quantity discount effect has been extensively considered. For example, Cha and Moon (2005) [18] studied a joint replenishment problem with quantity discount and developed two efficient heuristic algorithms to solve it. Choudhary and Shankar (2013) [19] developed an integrated model considering all-unit quantity discount for inventory lot-sizing, supplier selection, and carrier selection problem. Paul et al. (2014) [20] developed the JRP models for defective items with or without quantity discount, and proposed an effective heuristic algorithm to solve the models. Ongkunarul et al. (2016) [21] proposed a JRP model with resource constraints and defective items, and developed a genetic algorithm (GA) to determine the optimal reordering policy. Liu et al. (2018) [22] proposed a constrained joint replenishment and delivery model considering quantity discount, and designed a heuristic and hybrid Tabu search algorithm to solve it. Cui (2018) [23] proposed a novel multi-item joint replenishment problem with multiple-type discounts and developed a heuristic algorithm to find the optimal solution of the JRP model. Following these studies, our research focuses on a new JRP model, in which multiple non-instantaneous deteriorating items are joint replenished from multiple suppliers with different quantity discounts. Specifically, although the supplier selection problem is NP-hard, Chakraborty et al. (2020) [24] and Badi and Pamucar (2020) [25] proposed an excellent algorithm to address this complicated problem.

As various JRP models have received considerable critical attentions, many heuristic algorithms have been developed to solve them, such as the RAND algorithm (Cha and Moon, 2005 [18]), the hybrid genetic algorithm (HGA) (Moon et al., 2008 [5]), the differential evolution (DE) algorithm (Wang et al., 2012 [26]), the particle swarm optimization (PSO) algorithm (Kang et al., 2017 [27]), and the modified hybrid Tabu search (TS) algorithm (Liu et al., 2018 [22]), etc. Similarly, this paper follows this stream by developing a new and effective heuristic algorithm to solve the new proposed model.

3 The MFO algorithm and its applications

Recently, a novel swarm intelligence algorithm called the moth-flame optimization (MFO) has been proposed (Mirjalili, 2015 [7]). It has been then widely applied in different kinds of complicated optimization problems by many researchers, since its high efficiency and low memory usage endows the superiority over other algorithms. For example, Allam et al. (2016) [28] exploited the MFO algorithm in the parameter extraction process of the three diode models for the multi-crystalline solar module. It is noted that the MFO algorithm converges quickly but is easy to fall into a local optimum (Zhang et al., 2016 [8]). Savsani and Tawhid (2017) [29] designed an effective Non-dominated Sorting Moth-flame Optimization (NS-MFO) algorithm that is able to generate non-dominated solutions and true Parato front. They found that the NS-MFO outperforms both the GA and DE algorithm through numerical experiments. Fahad et al. (2019) [30] implemented a MFO algorithm to solve the robust routing problem by using the clustering techniques. Recently, several improved versions of the MFO algorithm have been proposed. For instance, Zhang et al. (2020) [31] proposed a new improved multi-objective MFO algorithm based on R-domination for cascade reservoirs operation. Lin et al. (2020) [32] developed an improved MFO algorithm for support vector machine prediction of the photovoltaic power generation. Cui et al. (2020) [33] proposed an improved MFO algorithm with adaptive Levy-Flight perturbations to minimize the specific fuel consumption of fighters. Inspired by the effectiveness and vast applications of the MFO algorithm, our study follows this stream by proposing an IMFO algorithm to extend its applications to a new JRP problem.

From the above literature review, the main contributions of this paper are as follows. (a) A new multi-supplier multi-item joint replenishment model for non-instantaneous deteriorating items with quantity discount is proposed. (b) An improved MFO algorithm is designed to solve the proposed model, obtaining results with satisfactory accuracy and robustness. (c) Effects of the key parameters are analyzed to help managers make better decisions on dealing with the selection of several suppliers and the joint replenishment of perishable products.

## 3. Formulation of the proposed model

### 3.1 Assumptions and notations

**Fig 1:**
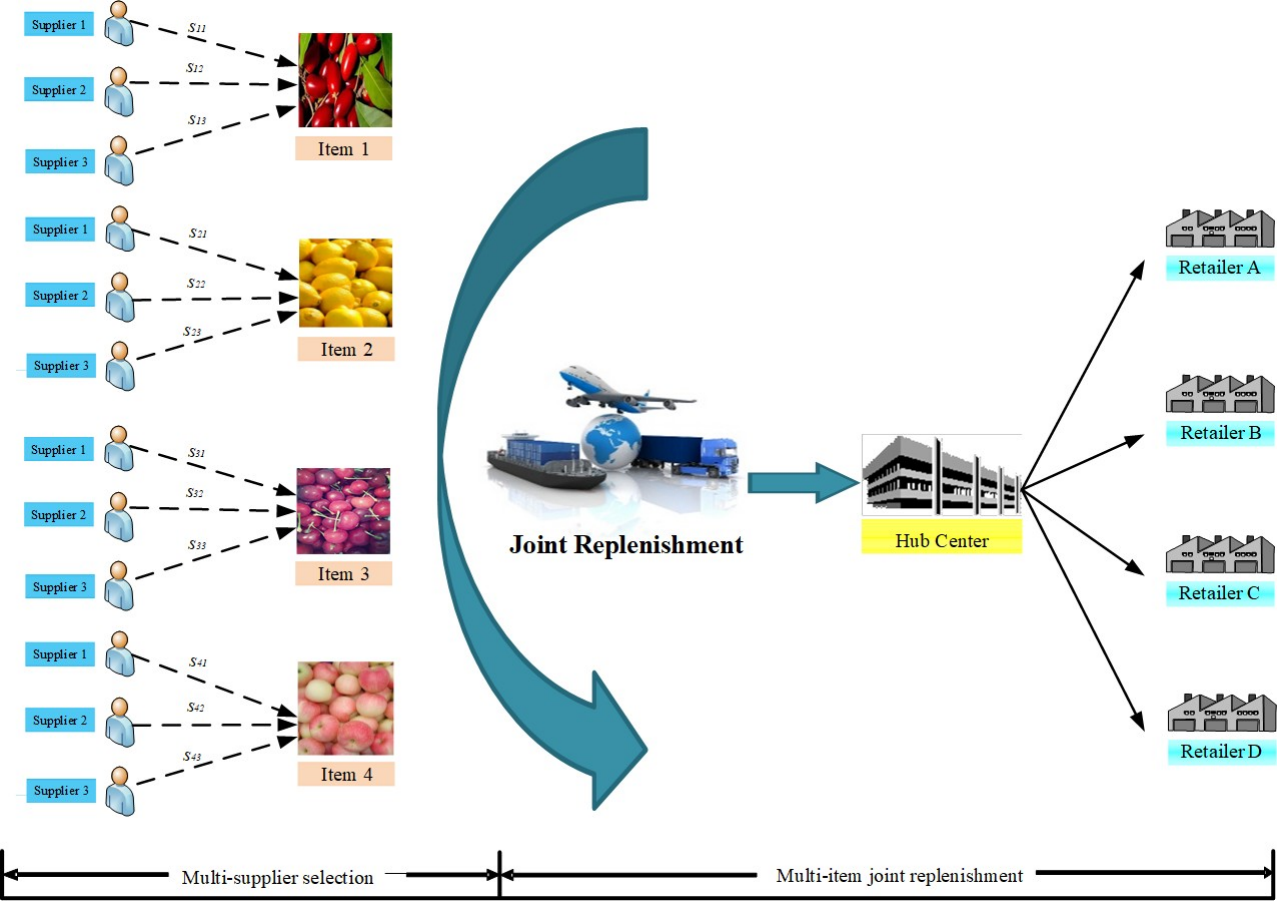
An example of multi-supplier multi-item joint replenishment for non-instantaneous deteriorating products.

A MS-JRNID problem involves the coordinated replenishment for several heterogeneous non-instantaneous deteriorating products under different quantity discount. The objective is to find a policy to minimize the total cost. Fig 1 presents a simplified example for illustrating the MS-JRNID network containing three suppliers and four perishable products in a supply chain. In the joint replenishment problem, a major ordering cost will be incurred whenever an order is placed. It is independent of the number of different items in the replenishment order. While a minor ordering cost, involving the packing charge, the preservation cost or order-processing cost, is incurred for each specific item. Obviously, the minor ordering cost may be different when replenishing from different suppliers.

The following assumptions are considered in our problem.

1. The demand rate of each item is constant and deterministic.
2. The replenishment lead time is zero and shortages are not permitted.
3. *t*_*di*_ is the fixed preservation period, in which no deterioration of item *i* occurs. After this period, item i deteriorates at a deterioration rate *θ*_*i*_, where 0<*θ*_*i*_ <1. There is no repair or replacement of the deteriorated items during the replenishment cycle.
4. The price of each item is dependent on the magnitude of the replenishment of each item from each supplier. All-units discount scheme is considered in this paper.
5. Each item can be purchased from only one supplier.
6. Under periodic review policy, all items’ replenishment is set on a basic cycle time *T*, and the replenishment *T*_*i*_ of Item *i* is *k*_*i*_ times of this basic cycle time, i.e., *T*_*i*_ = *k*_*i*_ *T*.
7. Every *T* time units, there is an order for at least one item.

The following notations in Table 1 are used to develop the model.

**Table 1:** Notations.

Parameter	Description
*n*	Number of items
*m*	Number of suppliers
*D*_*i*_	Demand rate of item *i*
*y*	Index of the price break
*h*_*i*_	Inventory cost of item *i* per unit per unit time
*I*_*1i*_*(t)*	Inventory level of item *i* at time *t* in Case 1
*S*	Major ordering cost which is incurred whenever an order is placed
*s*_*ij*_	Minor ordering cost if item *i* purchased from supplier *j* is included
*c*_*i*_	The fixed deterioration cost of item *i*
*Q*_*i*_	The order quantity of item *i* in each order
*P*_*ijy*_	Price of item *i* ordered from supplier *j* in the *y*th price break
*X*_*ij*_	Binary variable equal to 1 if item *i* is ordered from the *j*th supplier, otherwise it is 0 (decision variable)

### 3.2 Mathematical model

The non-instantaneous deteriorating items begin to decay after the fixed preservation period *t*_*di*_. Hence, there are two possible cases based on the value of *t*_*di*_ and *T*_*i*_: (1) *T*_*i*_ > *t*_*di*_ and (2) *T*_*i*_ ≤ *t*_*di*_.

Case 1: *T*_*i*_ > *t*_*di*_

Without loss of generality, we focus on a certain non-instantaneous deteriorating item *i*. During the time interval [0, *t*_*di*_], the inventory level decreases only due to the demand depletion. Then the item begins to decay and the inventory level drops to zero due to both the demand depletion and the deterioration during the time interval [*t*_*di*_, *T*].

Hence, the inventory level of item *i* could be described as follows:

$$\frac{dI_{1i}(t)}{dt}=\left\{ \begin{array}{l} -D_{i},\;0<t\leq t_{di}\\ -D_{i}-\theta_{i}I_{i}(t),\;t_{di}\leq t\leq T_{i} \end{array}\right..$$ (1)

With the boundary condition *I*_*1i*_(*T*_*i*_) = 0, solving the above equations, we get the solution of Eq (1) as follows:

$$I_{1i}(t)=\left\{ \begin{array}{l} \frac{D_{i}}{\theta_{i}}[e^{\theta_{i}\left(T_{i}-t_{di}\right)}-1]+D_{i}(t_{di}-t),\;0<t\leq t_{di}\\ \frac{D_{i}}{\theta_{i}}[e^{\theta_{i}(T_{i}-t)}-1],\;t_{di}\leq t\leq T_{i} \end{array}\right..$$ (2)

The order quantity for each order is then given by:

$$Q_{1i}=\frac{D_{i}}{\theta_{i}}[e^{\theta_{i}\left(T_{i}-t_{di}\right)}-1]+D_{i}t_{di}.$$ (3)

The average holding cost is:

$$HC_{1}=\sum_{i=1}^{n}\frac{h_{i}}{T_{i}}\int_{0}^{T_{i}}I_{1i}(t)dt=\sum_{i=1}^{n}\frac{D_{i}h_{i}}{k_{i}T}\{\frac{t_{di}^{2}}{2}+\frac{t_{di}}{\theta_{i}}[e^{\theta_{i}(T_{i}-t_{di})}-1]+\frac{1}{\theta_{i}^{2}}[e^{\theta_{i}(T_{i}-t_{di})}-\theta_{i}(T_{i}-t_{di})-1]\},$$ (4)

the average deterioration cost is:

$$DC_{1}=\sum_{i=1}^{n}\frac{c_{i}}{T_{i}}(Q_{1i}-D_{i}T_{i})=\sum_{i=1}^{n}\frac{c_{i}D_{i}}{\theta_{i}k_{i}T}[e^{\theta_{i}\left(T_{i}-t_{di}\right)}-\theta_{i}(T_{i}-t_{di})-1],$$ (5)

and the total ordering cost is:

$$OC_{1}=\frac{1}{T}(S+\sum_{i=1}^{n}\sum_{j=1}^{m}\frac{s_{i}X_{ij}}{k_{i}}).$$ (6)

Suppose that *C*_*1ij*_, which is a step function of *T* and *k*_*i*_, is the unit purchasing cost function of item *i* from *j*th supplier. For the all-units quantity discount scheme, *C*_*1ij*_ is represented as follows:

$$C_{1ij}(k_{i},T)=\left\{ \begin{array}{l} p_{ij1}\;,\;Q_{1i}<q_{ij1}\\ p_{ij2}\;,\;q_{ij1}\leq Q_{1i}<q_{ij2}\\ \vdots \;,\;\vdots \\ p_{ijy\prime }\;,\;q_{ij(y'-1)}\leq Q_{1i}<q_{ijy'}\\ \vdots \;,\;\vdots \\ p_{ij(y-1)}\;,\;q_{ij(y-2)}\leq Q_{1i}<q_{ij(y-1)}\\ p_{ijy}\;,\;q_{ij(y-1)}\leq Q_{1i}<q_{ijy} \end{array}\right.,$$

where y represents the price discount categories. The preceding analysis shows that $P_{ij1}>P_{ij2}>\cdots >P_{ijy^{\prime }}>\cdots >P_{ijy}$. Furthermore, the marginal growth of quantity discount decreases normally. Therefore, we can obtain the following inequality:

$$\frac{P_{ij(y-1)}-P_{ijy}}{P_{ijy}-P_{ij(y-1)}}\leq \frac{P_{ij(y-2)}-P_{ij(y-1)}}{P_{ij(y-1)}-P_{ij(y-2)}}\leq \cdots \leq \frac{P_{ij(y'-1)}-P_{ijy'}}{P_{ijy'}-P_{ij(y'-1)}}\leq \cdots \leq \frac{P_{ij2}-P_{ij3}}{P_{ij3}-P_{ij2}}\leq \frac{P_{ij1}-P_{ij2}}{P_{ij2}-P_{ij1}}$$

As a result, the average purchase cost is denoted by:

$$PC_{1}=\sum_{i=1}^{n}\sum_{j=1}^{m}\frac{X_{ij}C_{1}{}_{ij}Q_{1i}}{k_{i}T}=\sum_{i=1}^{n}\sum_{j=1}^{m}\frac{X_{ij}C_{1ij}D_{i}}{\theta_{i}k_{i}T}[e^{\theta_{i}\left(k_{i}T-t_{di}\right)}+\theta_{i}t_{di}-1]$$ (7)

Therefore, the total cost is:

$$\begin{array}{l} TC_{1}(k_{i},T,X_{ij})=\sum_{i=1}^{n}\frac{c_{i}D_{i}}{\theta_{i}k_{i}T}[e^{\theta_{i}\left(T_{i}-t_{di}\right)}-\theta_{i}(T_{i}-t_{di})-1]+\sum_{i=1}^{n}\frac{h_{i}D_{i}}{k_{i}T}[(\frac{t_{di}}{\theta_{i}}+\frac{1}{\theta_{i}^{2}})e^{\theta_{i}\left(T_{i}-t_{di}\right)}\\ \;-\frac{1}{\theta_{i}}(T_{i}-t_{di})-\frac{t_{di}}{\theta_{i}}+\frac{t_{di}^{2}}{2}-\frac{1}{\theta_{i}^{2}}]+\frac{1}{T}(S+\sum_{i=1}^{n}\sum_{j=1}^{m}\frac{s_{i}X_{ij}}{k_{i}})+\\ \;\sum_{i=1}^{n}\sum_{j=1}^{m}\frac{X_{ij}C_{1ij}D_{i}}{\theta_{i}k_{i}T}[e^{\theta_{i}\left(k_{i}T-t_{di}\right)}+\theta_{i}t_{di}-1] \end{array}$$ (8)

where

$$\sum_{j=1}^{m}X_{ij}=1,for\;i=1,2,\ldots ,n$$

$$C_{1ij}(k_{i},T)=p_{ijy},for\;q_{ijy}\leq Q_{1i}<q_{ij(y+1)};$$

$$Q_{1i}=\frac{D_{i}}{\theta_{i}}[e^{\theta_{i}\left(T_{i}-t_{di}\right)}-1]+D_{i}t_{di}$$

$$\begin{array}{l} k_{i}\in N^{n}\;\mathrm{and}\;k_{i}=1\;\mathrm{for}\;\mathrm{some}\;1\leq i\leq n;\\ 0<T<1; \end{array}$$

Case 2: *T*_*i*_ ≤ *t*_*di*_

In this case, the product replenishment cycle is less than or equal to the deterioration free time of the product, implying that the retailer can sell out all products before they start to decay. Therefore, we do not need to consider the deterioration. The model turns into the traditional inventory model and the total cost per unit time is given by:

$$TC_{2}(k_{i},T,X_{ij})=\frac{T}{2}\sum_{i=1}^{n}k_{i}D_{i}h_{i}+\frac{1}{T}(S+\sum_{i=1}^{n}\sum_{j=1}^{m}\frac{s_{i}X_{ij}}{k_{i}})+\sum_{i=1}^{n}\sum_{j=1}^{m}X_{ij}C_{2ij}D_{i}$$ (9)

where,

$$\sum_{j=1}^{m}X_{ij}=1,for\;i=1,2,\ldots ,n$$

$$C_{2ij}(k_{i},T)=p_{ijy},\;for\;q_{ij(y-1)}\leq D_{i}k_{i}T<q_{ijy}$$

$$\begin{array}{l} k_{i}\in N^{n}\;\mathrm{and}\;k_{i}=1\;\mathrm{for}\;\mathrm{some}\;1\leq i\leq n;\\ 0<T<1; \end{array}$$

Combining Case 1 and Case 2, along with T_i_ = *k*_*i*_*T*, the average total cost is:

$$\begin{array}{l} \min TC(k_{i},T,X_{ij})=\frac{S}{T}+\sum_{i=1}^{n}\frac{\left[1+sign(k_{i}T-t_{di})\right]}{2}\{\frac{c_{i}D_{i}}{\theta_{i}k_{i}T}[e^{\theta_{i}\left(k_{i}T-t_{di}\right)}-\theta_{i}(k_{i}T-t_{di})-1]\\ \;+\frac{h_{i}D_{i}}{k_{i}T}[(\frac{t_{di}}{\theta_{i}}+\frac{1}{\theta_{i}^{2}})e^{\theta_{i}\left(k_{i}T-t_{di}\right)}-\frac{1}{\theta_{i}}(k_{i}T-t_{di})-\frac{t_{di}}{\theta_{i}}+\frac{t_{di}^{2}}{2}-\frac{1}{\theta_{i}^{2}}]\\ \;+\sum_{j=1}^{m}\frac{s_{i}X_{ij}}{k_{i}T}+\sum_{j=1}^{m}\frac{X_{ij}C_{ij}D_{i}}{\theta_{i}k_{i}T}[e^{\theta_{i}\left(k_{i}T-t_{di}\right)}+\theta_{i}t_{di}-1]\}\\ \;+\sum_{i=1}^{n}\frac{\left[1-sign(k_{i}T-t_{di})\right]}{2}(\frac{k_{i}T}{2}D_{i}h_{i}+\sum_{j=1}^{m}\frac{s_{i}X_{ij}}{k_{i}T}+\sum_{j=1}^{m}X_{ij}C_{ij}D_{i}) \end{array},$$ (10)

where

$$\begin{array}{l} k_{i}\in N^{n}\;\mathrm{and}\;k_{i}=1\;\mathrm{for}\;\mathrm{some}\;1\leq i\leq n;\\ 0<T<1;\\ \sum_{j=1}^{m}X_{ij}=1,for\;i=1,2,\ldots ,n;\\ C_{ij}(k_{i},T)=p_{ijy},for\;q_{ijy}\leq Q_{{}_{i}}<q_{ij(y+1)};\\ where\;Q_{i}=\frac{\left[1+sign(k_{i}T-t_{di})\right]}{2}\{\frac{D_{i}}{\theta_{i}}[e^{\theta_{i}\left(T_{i}-t_{di}\right)}+\theta_{i}t_{di}-1]\}+\frac{\left[1-sign(k_{i}T-t_{di})\right]}{2}D_{i}k_{i}T \end{array}$$

## 4. The solution method

### 4.1 The original Moth-Flame Optimization (MFO) algorithm

The MFO is a novel swarm intelligence optimization algorithm originally presented by Mirjalili (2015) [6]. It is a nature-inspired optimization technology that simulates the transverse orientation mechanism of moths.

In the transvers orientation, the moth flight process is performed with a fixed angle to the moon. Because the moon is far away from the moth, the transverse orientation guarantees flying in a straight line. In reality, moths usually fly spirally around the artificial light. Because once moths see the artificial light, they will try to maintain a similar angle with this light to fly in a straight line. However, such light is very close with respect to the moon, thus moths fly in a spiral path. Moths are deceived by artificial light due to the transverse orientation inefficiency. The transverse orientation inefficiency confirms that it is available only for flying a long distance in a straight path.

The moths have spiral flight around the artificial light keeping similar angle with it. The moths’ positions are updated with their spiral flights. And in the optimization process, the spiral flight space must be within the search range. In the MFO, decision variables to be evaluated are the moths’ positions in the space. The solutions are the moths whose flights may be one, two, three or hybrid dimensions. Based on a swarm algorithm, the moths’ positions can be represented by the matrix as follows:

$$M=\left[\begin{array}{cccc} m_{1,1} & m_{1,2} & \cdots & m_{1,d}\\ m_{2,1} & m_{2,2} & \cdots & m_{2,d}\\ \vdots & \vdots & \ddots & \vdots \\ m_{n,1} & m_{n,2} & \cdots & m_{n,d} \end{array}\right]$$ (11)

$$\mathrm{OM}={\left[\begin{array}{cccc} OM_{1} & OM_{2} & \cdots & OM_{n} \end{array}\right]}^{T}$$ (12)

where n is the number of moths, and d is the problem dimensions. Moreover, Eq (11) denotes positions of the moths. Then the corresponding fitness functions are calculated. The vector OM stores the corresponding fitness values of n moths.

Another key component of the MFO is the flames positions defined as:

$$F=\left[\begin{array}{cccc} F_{1,1} & F_{1,2} & \cdots & F_{1,d}\\ F_{2,1} & F_{2,2} & \cdots & F_{2,d}\\ \vdots & \vdots & \ddots & \vdots \\ F_{n,1} & F_{n,2} & \cdots & F_{n,d} \end{array}\right]$$ (13)

It is shown that the moths and the flames have the same search space dimensions. As for the flames, the fitness values are represented as follows:

$$\mathrm{OF}={\left[\begin{array}{cccc} OF_{1} & OF_{2} & \cdots & OF_{n} \end{array}\right]}^{T}$$ (14)

Moths and flames are both solutions, but are different in the way of being treated and updated. Moths represent the bodies moves in the search space, while flames represent the best positions acquired at present. Furthermore, the moths search and update the variables to be optimized, ensuring that moths will never miss their optimal solution. The MFO can be modelled in three main functions as follows:

MFO=(I,P,K) (15)

where: *I* is a function responsible for both generating random population of moths and calculating the corresponding fitness function; *P* is the main function of the optimizer that receives the matrix of moths and gives its updated one; and *K* is a function that verifies the criterion achievement, either true or false.

The moth’s position is updated according to the flame as follows:

$$M_{i}=S(M_{i},F_{j})$$ (16)

where *M*_*i*_ is the ith moth, *F*_*j*_ is the *j*th flame and *S* is the spiral function.

In the original MFO, the moth’s position is updated with the logarithmic spiral function. The characteristics of the spiral flight mechanism are as follows:

1. The starting point of spiral flight is the moth.
2. The endpoint of spiral flight is the flame position.
3. The spiral flight space must be within the search range.

For updating the position of each moth and simulating the flight mode of the moth, the MFO are as follows:

$$S(M_{i},F_{j})=D_{i}e^{bt}\cos(2\pi t)+F_{j}$$ (17)

where *b* is the spiral flight shape, *t* is a random number in range [–1, 1], and *D*_*i*_ represents the distance between the *i*th moth and the *j*th flame. In the equation, the next position of the moth is related to the flame. The spiral flight coefficient *t* defines how much the moth’s updated position is close to the flame (*t* = -1 indicates the closest position while *t* = 1 indicates the farthest position). Therefore, it can be assumed that there is a super-ellipse in all directions around the flame, and the moth’s updated position will be in this space. Because the moth’s position and flame structure are updated with the moth’s spiral flight, the spiral flight is the main function of the MFO algorithm.

Thereby, *D*_*i*_ can be expressed as follows:

$$D_{i}=|M_{i}-F_{j}|$$ (18)

For improving the local mining capacity of the moth in the later iteration, it can decrease the number of flames during each iteration in an adaptive linear way. It can be expressed as follows:

$$\mathrm{flame}\;\mathrm{no}=\mathrm{round}(N‐l\frac{N-1}{T})$$ (19)

where *l* is the current iteration times, *N* is the maximum number of flames and *T* denotes the maximum iteration times.

### 4.2 The improved MFO algorithm

In order to enhance the ability of the MFO and to avoid falling into a local optimum, the improvements of the MFO algorithm are executed in the following two aspects.

(1) The original MFO algorithm applies the logarithmic spiral function to simulate the moth’s spiral flight path. However, there exist other spiral functions, such as the Archimedean spiral, the Euler spiral, Hyperbolic spiral, the Fibonacci spiral, and the Fermat’s spiral etc. After a large number of trials for comparing the optimization performance of different spiral functions, the optimal spiral function could be chosen to simulate the moth’s spiral flight path. The test results show that the MFO algorithm with hyperbolic spiral function has the ability to obtain a set of solutions with good convergence and strong distribution in our problem. Therefore, the optimization accuracy and speed could be improved.

(2) Inspired by the Levy-Flight, we propose a MFO algorithm based on it. Because the Levy-Flight has a fast-growing variance, it can be more effective than the brown random motion in a large-scale searching process. In the Levy-Flight strategy, short-distance walking with small step size and occasional large-step walking are alternate. Therefore, on the one hand, some individuals search near the current optimal position to accelerate the local exploitation; on the other hand, the other part of individuals can search in the space far enough from the current optimal position to avoid all individuals falling into the local optimum. Thus, it can escape from the local optimum and improve the global search capability.

#### 4.2.1 The hyperbolic spiral function

**Fig 2:**
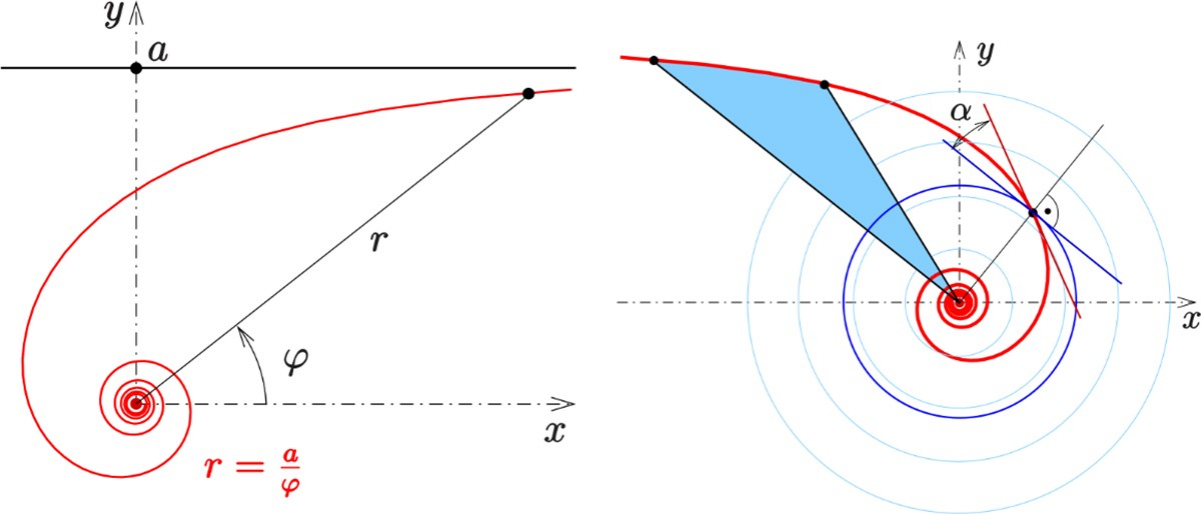
Hyperbolic spiral. (a) Hyperbolic spiral: $r=\frac{a}{\varphi }$ (b) Definition of sector (light blue) and polar slope angle.

As shown in Fig 2(A), the reciprocal spiral uses the polar equation *r* = *a/ψ*, where, *r* and *ψ* are the radius and azimuthal angle in a polar coordinate system, respectively. As *ψ* increases, the spiral winds around the origin and moves closer to it. It winds faster and faster around as it approaches the pole. Fig 2(B) shows definitions of the sector (light blue) and the polar slope angle. Taking the pole as the center of inversion, the hyperbolic spiral inverts to the spiral of Archimedes.

Applying the hyperbolic spiral function to simulate the moth’s spiral flight path, the Eq (17) can be improved as follows:

$$S(M_{i},F_{i})=D_{i}\frac{\cos(2\pi t)}{t}+F_{i},$$ (20)

where

*S* is the hyperbolic spiral function (movement).

*t* is the random number in range [–1, 1], and defines how much the next position of moth should be close to the flame. *t* = -1 indicates the closest position to the flame while *t* = 1 indicates the farthest position.

#### 4.2.2 Levy-Flight algorithm

The Levy-Flight is a probability distribution proposed by French mathematician Paul Pierre Levy. It is a Markov process, where its distribution follows the heavy probability distribution. Inspired by the Levy-Flight, many scholars applied the Levy-Flight strategy to improve their algorithms, which effectively improve the quality of the solution.

The Levy-Flight mechanism can be defined as follows:

$$\mathrm{Levy}(s)∼{\left|s\right|}^{-\lambda },\;1<\lambda <3$$ (21)

where s is the random moving step size, and λ is the exponential parameter. Eq (21) is the heavy tail probability distribution, which is hard to performance through the simple programming language. Therefore, when calculating the Levy-Flight searching distance, it is common to apply the following Eq (22) to simulate the Levy-Flight path proposed by Mantegna.

$$s={\mu /\left|v\right|}^{1/\beta }$$ (22)

where s is the Levy-Flight distance, and parameter β is defined in range (0, 2), and normally set as β = 1.5. Both μ and ν represent normal distribution showing in the Eq (23), and the standard deviation corresponding to the Eq (23) satisfies to the Eq (24).

$$\mu ∼N(0,\sigma_{\mu }^{2}),\;v∼N(0,\sigma_{v}^{2})$$ (23)

$$\sigma_{\mu }={\left\{\frac{\Gamma (1+\beta )\sin(\pi \beta /2)}{\Gamma [\left(1+\beta \right)/2]2^{\left(\beta -1\right)/2}\beta }\right\}}^{1/\beta },\;\sigma_{v}=1$$ (24)

As a result, the Levy-Flight path can be calculated through Eq (22) to Eq (24).

Applying the Eq (25) instead of the Eq (18) in the original MFO algorithm can express the moth’s Levy-Flight path as follows:

$$D_{i}=\left|F_{i}-M_{i}\right|\mathrm{Levy}(\lambda )$$ (25)

According to the population aggregation status, the Levy-Flight disturbance strategy can adaptively adjust the perturbation probability to enhance the ability of the IMFO algorithm to escape from local optimal solutions and realize the minimum total cost.

### 4.3 The algorithm design

Based on the above discussions, the procedure of our proposed IMFO algorithm for solving the MS-JRNID model is as follows:

Step 1: Determine the problem dimensions, the searching scope and the criterions of parameters *K*_*i*_, *T*, *X*_*ij*_ to be optimized.
Step 2: Determine the fitness function of the MFO algorithm. This study chooses Eq (10) as the fitness function.
Step 3: Initialize the parameters. Set the number of iterations, the population size, the search space and the flame number. Set the current iteration times *l* = 0.
Step 4: Calculate the fitness value of individuals. Find the current optimal moth position and keep it as the flame fitness value matrix. Judge whether the maximum iteration number is reached, if reached, go to Step 8, otherwise, execute Step 5.
Step 5: Execute iterative processes. Update the flame number based on Eq (19), calculate the distance between the moth and the flame, and update the moth position and the flame position based on the Eq (20) and Eq (25).
Step 6: Calculate the fitness value of individuals. Keep the moth position and the flame position based on the Eq (12) and Eq (14).
Step 7: Find the current optimal moth position. If the current position is better than the previous position, keep the current flame position as the optimal position. Judge whether the maximum iteration number is reached or not, if reached, go to the Step 8, otherwise, set *l* = *l* + 1 and return to the Step 5.
Step 8: Output the optimal individual and global extremum, that is, the final flame position *K*_*i*_, *T*, *X*_*ij*_ and corresponding fitness value. The process ends.

**Fig 3:**
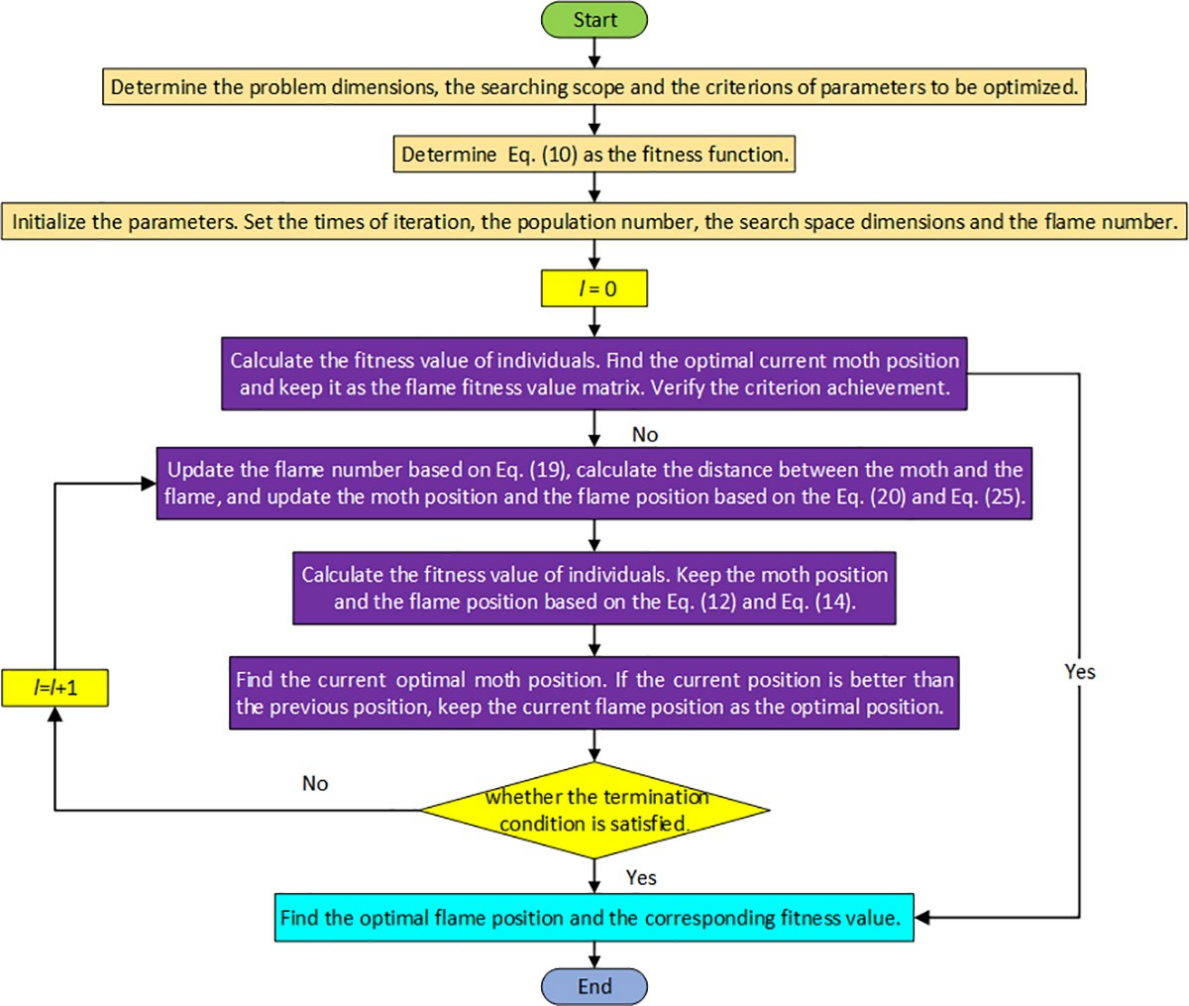
Flow chart of IMFO for proposed MS-JRNID.

Fig 3 presents the flow chart of the developed IMFO algorithm. After executing the above steps, the optimal strategy to Eq (10) is given and corresponding minimal cost TC* can be obtained. Then (*k*_1_*, *k*_2_*, *…*, *k*_n_*, T*, *X*_11_*, *X*_12_*, *…*, *X*_ij_*, *…*, *T*_nm_*) can be regarded as the optimal solution of the proposed model.

## 5. Numerical experiments

This section aims to evaluate the performance of the proposed approach and MS-JRNID. In order to verify the competitive performance of the IMFO algorithm, it is compared with other five meta-heuristic algorithms, named Genetic Algorithm (GA) (Deb et al. 2002) [34], Grey Wolf Optimizer Algorithm (GWO) (Mirjalili et al. 2014) [35], Fruit-fly Optimization Algorithm (FOA) (Pan, 2012) [36], Particle Swarm Optimization (PSO) Algorithm (Shi, 2001) [37] and original Math-Flame Optimization Algorithm (MFO) (Mirjalili. 2015) [7]. Furthermore, sensitive analyses of the parameters are performed to find the optimal results of the proposed model.

The parameters of the numerical example come from the related works in literature (Moon et al. 2008 [5], Cui et al. 2016 [38], Liu et al. 2018 [22]). Since they did not consider the deterioration of items, we introduce the parameters *θ*_*i*_, *t*_*di*_, and *c*_*i*_, which represent the deterioration rate, the non-deterioration time and the deterioration cost, respectively. The values of parameters are shown in the Table 2. Moreover, the quantity discount strategies provided by each supplier are shown in the Table 3.

**Table 2:** Parameter settings of non-instantaneous deteriorating items (n = 10).

Item	1	2	3	4	5	6	7	8	9	10
***s***_***i1***_	5.0	19.4	9.5	8.5	2.2	8.2	10.6	4.0	20.0	16.0
***s***_***i2***_	5.0	19.2	9.0	9.2	2.0	8.0	10.4	4.2	24.0	15.0
***s***_***i3***_	5.2	19.4	8.4	9.2	2.4	7.8	11.2	4.2	24.0	18.0
***D***_***i***_	600	900	2400	12000	18000	3000	2500	180	50	146
***h***_***i***_	0.5	1.94	0.95	0.85	0.22	0.82	1.06	0.4	2.0	1.6
***c***_***i***_	3	11.64	5.7	5.1	1.32	4.92	6.36	2.4	12	9.6
***θ***_***i***_	0.02	0.02	0.02	0.02	0.02	0.02	0.02	0.02	0.02	0.02
***t***_***di***_	0.0411	0.0822	0	0.0411	0.411	0.0822	0.0822	0	0.0411	0.822
***S***	10									

**Table 3:** The quantity discount strategies provided by each supplier (* represent no price discount).

*j*	*q*_*ijy*_	*C*_*1j*_	*C*_*2j*_	*C*_*3j*_	*C*_*4j*_	*C*_*5j*_	*C*_*6j*_	*C*_*7j*_	*C*_*8j*_	*C*_*9j*_	*C*_*10j*_
1	*Q*_*i*1_<150	2.5	9.7	4.75	4.25*	1.1	4.1	5.3	2.0*	10.0*	8.0
	150≤*Q*_*i*1_<300	2.2	9.6	4.4	-	1.05	3.9	5.1	-	-	7.0
	*Q*_*i*1_≥300	2.1	9.4	4.1	-	0.95	3.7	4.8	-	-	6.0
2	*Q*_*i*2_<200	2.5	9.6*	4.5	4.6	1.0*	4.0	5.2*	2.1	12.0	7.5*
	200≤*Q*_*i*2_<400	2.2	-	4.2	4.25	-	3.8	-	2.05	8.0	-
	*Q*_*i*2_≥400	1.9	-	4.0	4.1	-	3.5	-	1.95	6.0	-
3	*Q*_*i*3_<300	2.6	9.7	4.2*	4.6	1.2	3.9	5.6	2.1	12.0	9.0
	300≤*Q*_*i*3_<500	2.3	9.5	-	4.3	1.1	3.8	5.0	1.95	9.0	6.0
	*Q*_*i*3_≥500	2.0	9.3	-	4.0	0.9	3.4	4.6	1.90	5.0	5.0

### 5.1 Comparison between GA, FOA, PSO, GWO, MFO and IMFO

In this subsection, we compare the performances of the proposed IMFO algorithm with other algorithms. Among the meta-heuristic methods, the GA is a stochastic search algorithm following the mechanism of natural selection and “survival of the fittest”, and has been widely used in production and operations management problems (May et al. 2015) [39]. The FOA has been shown to be an efficient and effective algorithm for many JRPs (Wang, Shi, and Liu 2015) [40]. The PSO is a well-known method that optimizes a problem by iteratively trying to improve a candidate solution with regard to a given measure of quality, the experience of which is very convenient for testing other algorithms. The GWO algorithm mimics the leadership hierarchy and hunting mechanism of grey wolves in nature, it is a new meta-heuristic inspired by grey wolves. Therefore, GA, FOA, PSO, GWO and MFO are selected for comparisons with the proposed solution method in this paper.

In order to facilitate a direct comparison of the performance of different algorithms, the control parameters of GA, FOA, PSO, GWO, MFO are selected based on pilot experiments from Deb et al. (2002) [34]; Pan (2012) [36]; Shi (2001) [37]; Mirjalili et al. (2014) [35]; and Mirjalili (2015) [7] respectively. For all algorithms, the population size is kept to be 30. The iteration termination condition is to stop after 500 generations. Each algorithm is run for 20 times with different seeds. All experiments are performed by MATLAB in the computer with CPU@2.27GHz and 2.00GB RAM.

**Fig 4:**
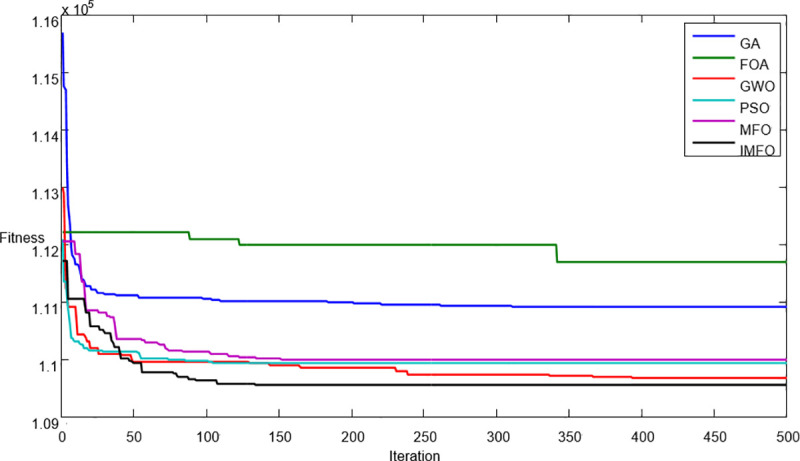
Convergence graphs of average best-so-far solutions obtained by GA, FOA, GWO, PSO, MFO, and IMFO.

Under the given values of parameters, we can obtain the computational results of different algorithms shown in Table 4. The proposed IMFO can find a better solution compared to other algorithms. To display the convergence features of each algorithm in searching processes, the average best-so-far solution progress over iterations number is shown in Fig 4.

**Table 4:** Experimental results of six algorithms.

Algorithm	Optimal supplier selection strategy	Optimal replenishment strategy (*k*_i_*)	Optimal basic replenishment cycle (T*)	Optimal total cost (TC*)	IMFO better than other algorithms (%)
GA	2, 1, 2, 3, 3,	15, 8, 4, 1, 1,	0.0442	109627.43	0.16
	3, 3, 1, 2, 2	4, 5, 6, 94, 16			
FOA	1, 1, 1, 1, 1,	1, 1, 4, 1, 1,	0.0204	110952.53	1.35
	3, 3, 2, 1, 1	6, 2, 2, 2, 1			
GWO	2, 1, 2, 3, 3,	16, 8, 4, 1, 1,	0.0417	109465.34	0.01
	3, 3, 1, 1, 2	4, 5, 7,17,6			
PSO	2, 1, 2, 3, 3,	16, 8, 4, 1, 1,	0.0417	109521.43	0.06
	3, 3, 1, 2, 3	4, 5, 7, 93, 49			
MFO	2, 2, 2, 3, 3,	16, 3, 4, 1, 1,	0.0420	109506.34	0.05
	3, 3, 1, 1, 1	4, 5, 7, 14, 48			
IMFO	2, 1, 2, 3, 3,	12, 6, 3, 1, 1,	0.0555	109452.70	-
	3, 3, 1, 1, 2	3, 4, 5, 10, 6			

Based on the computation results shown in Table 4 and Fig 4, the following results can be derived.

1. The basic cycle time *T* and the optimal replenishment strategy *k*_*i*_ show different features under different algorithms.
2. The optimal total cost of the IMFO algorithm is smaller than that of GA, FOA, GWO, PSO, and MFO. The average percentage savings in total costs provided by the IMFO are 0.01–1.35 when compared to other five algorithms.
3. As for the convergence features of each algorithm, it can be seen that GA, PSO and GWO converge quickly in the early stage of the search process, but converge slowly in the late stage of the search process, while the IMFO finds the minimum total cost at the end of iterations. Therefore, the IMFO shows a competitive advantage over other algorithms.

### 5.2 Sensitivity analyses

**Fig 5:**
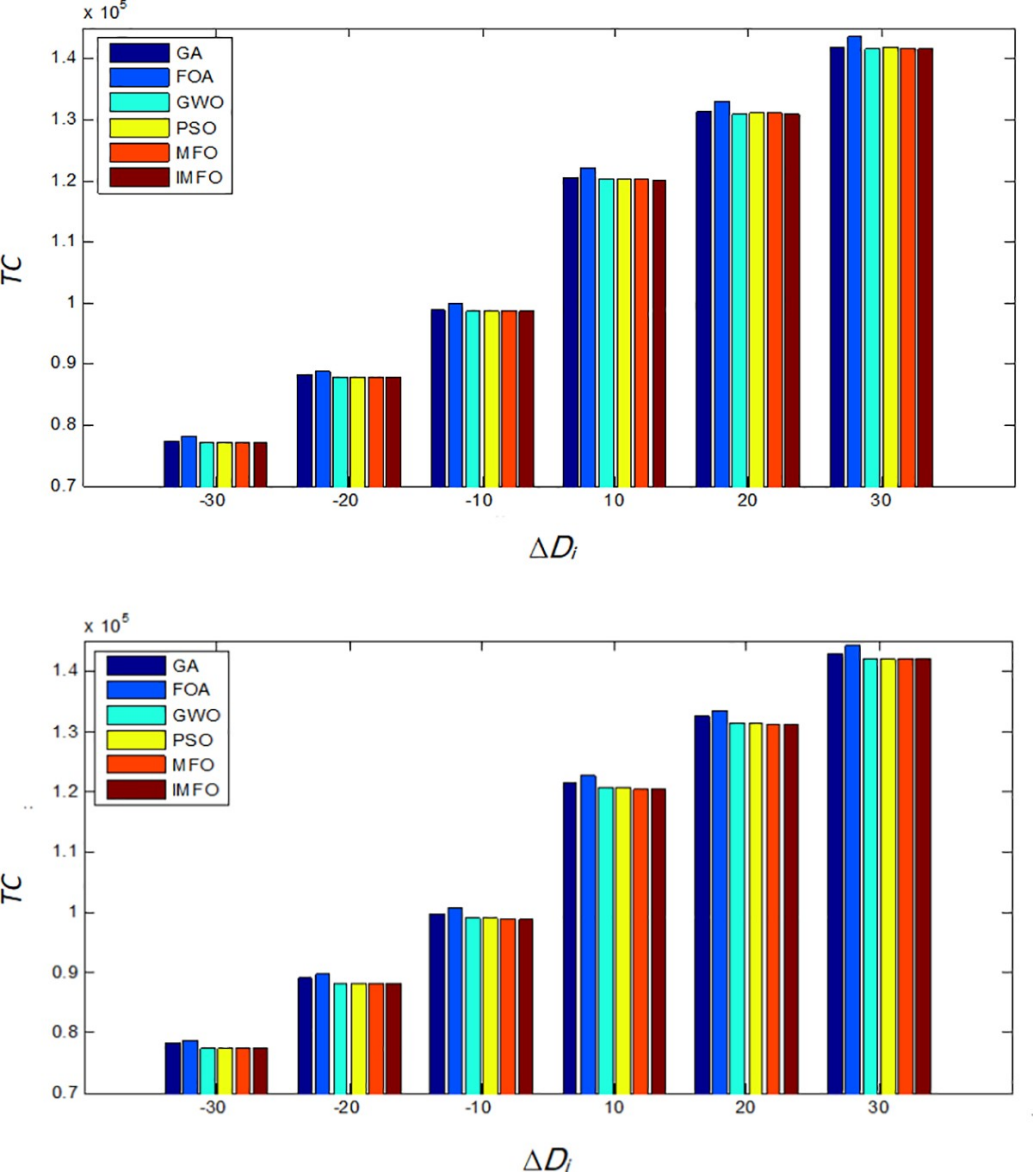
When *D*_*i*_ changes, the comparison results of *TC*. (a) The comparison of the minimum value of *TC*. (b) The comparison of the average value of *TC*.

**Fig 6:**
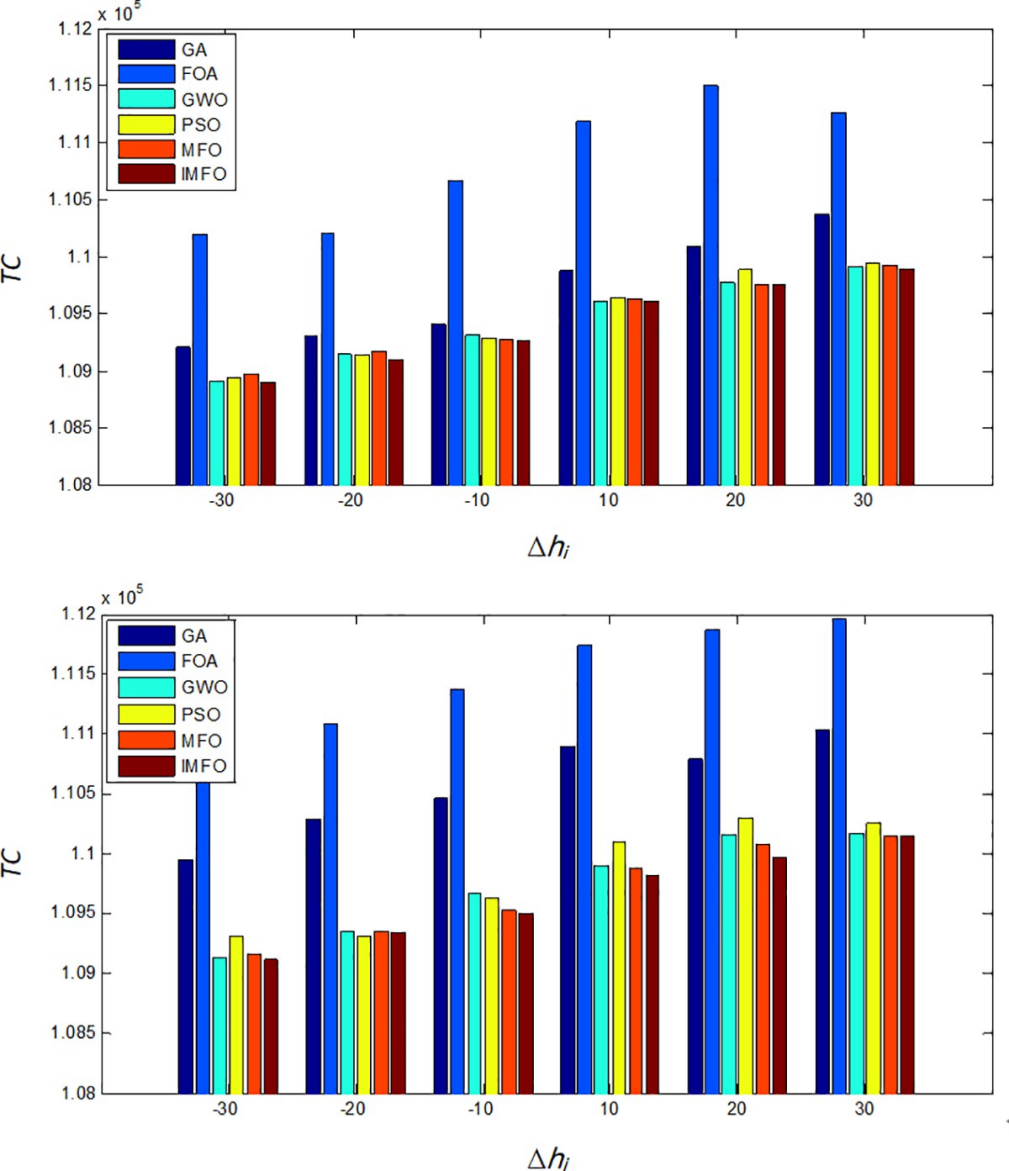
When *h*_*i*_ changes, the comparison results of *TC*. (a) The comparison of the minimum value of *TC*. (b) The comparison of the average value of *TC*.

**Fig 7:**
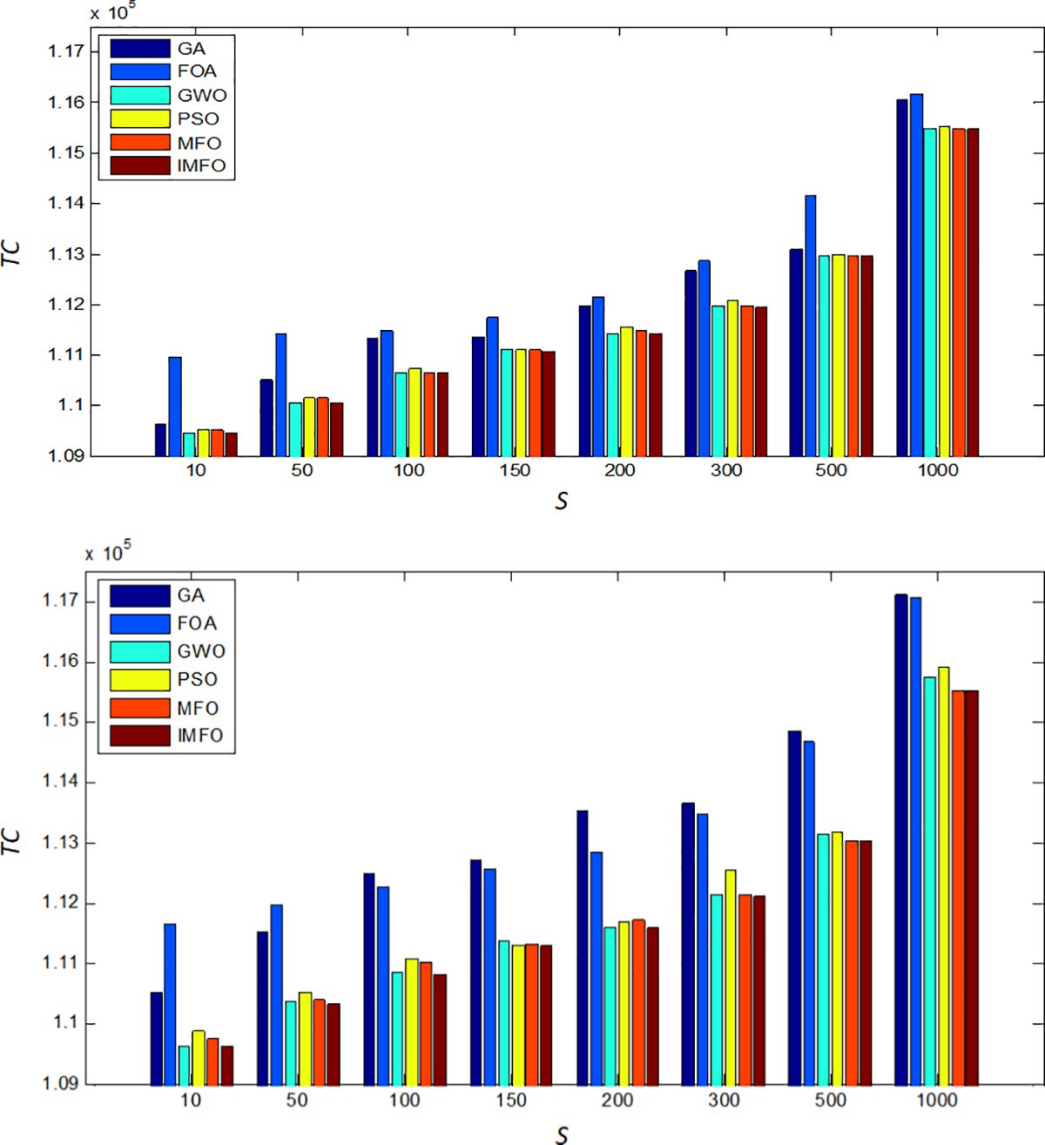
When S changes, the comparison results of TC. **(a)** The comparison of the minimum value of *TC*. (b) The comparison of the average value of TC.

In this subsection, we explore some managerial insights based on sensitivity analyses of the parameters. We investigate the effects of changes in system parameters *D*_*i*_ and *h*_*i*_ on the performances of the six algorithms (GA, FOA, GWO, PSO, MFO, and IMFO). Sensitive analyses are performed by changing each of the parameters by -30%, -20%, -10%, 10%, 20% and 30%, taking one parameter at a time and keeping the remaining parameters unchanged. Moreover, we compare the performances of the six algorithms with different values of *S*. The results are shown in Figs 5–7.

Based on the computation results shown in Figs 5–7, the following observations can be derived.

1. When the values of parameter *D*_*i*_, *h*_*i*_ and *S* increases, the optimal total costs of each algorithm will also increase. In addition, when *D*_*i*_ changes, the minimum total cost of IMFO are slightly less than the cost obtained from GWO, PSO, and MFO, and much less than those from GA and FOA.
2. With the changes of *h*_*i*_, the minimum and average total cost obtained from FOA is the highest, while the solution of the IMFO is the best.
3. It can be found that when S changes, as for the results performance of the average total cost, the IMFO is more competitive.

Furthermore, when *D*_*i*_, *h*_*i*_, and *S* change, the optimal supplier mixture strategy (*X*_*ij*_), the optimal replenishment strategy (*k*_i_*), the optimal basic replenishment cycle (T*), and the optimal system total cost (TC*) are shown in Tables 5–7.

**Table 5:** When Di changes, the optimal solution obtained by the IMFO algorithm.

Δ*D*_*i*_(%)	The optimal supplier mixture strategy	The optimal replenish strategy (*k*_i_*)	The optimal basic replenish cycle (T*)	The optimal total cost (TC*)	ΔTC*(%)
-30	2, 2, 2, 3, 3, 3, 3, 1, 1, 2	16, 3, 4, 1, 1,	0.05952	77097.88	-29.56
		4, 5, 6, 11, 7			
-20	2, 1, 2, 3, 3, 3, 3, 1, 1, 1	16, 8, 4, 1, 1,	0.05208	87920.94	-19.67
		4, 5, 6, 12, 25			
-10	2, 1, 2, 3, 3, 3, 3, 1, 1, 2	16, 8, 4, 1, 1,	0.04627	98680.53	-9.84
		4, 5, 7, 13, 7			
10	2, 1, 2, 3, 3, 3, 3, 1, 1, 3	16, 8, 4, 1, 1,	0.03827	120206.26	9.82
		4, 5, 8, 14, 48			
20	2, 1, 2, 3, 3, 3, 3, 1, 1, 3	12, 6, 3, 1, 1,	0.04684	130985.67	19.67
		3, 4, 1, 11, 36			
30	2, 1, 2, 3, 3, 3, 3, 1, 1, 3	12, 6, 3, 1, 1,	0.04273	141660.87	29.43
		3, 4, 6, 12, 37			

**Table 6:** When *D*_*i*_ changes, the optimal solution obtained by the IMFO algorithm.

Δ*h*_*i*_(%)	The optimal supplier mixture strategy	The optimal replenish strategy (*k*_i_*)	The optimal basic replenish cycle (T*)	The optimal total cost (TC*)	ΔTC*(%)
-30	2, 1,2, 3, 3, 3, 3, 1, 1, 3	12, 6, 3, 1, 1,	0.05553	108896.76	-0.51
		3, 4, 6, 12, 60			
-20	2, 1,2, 3, 3, 3, 3, 1, 1, 3	12, 6, 3, 1, 1,	0.05623	109097.15	-0.32
		3, 4, 6, 11, 59			
-10	2, 1,2, 3, 3, 3, 3, 1, 1, 3	12, 6, 3, 1, 1,	0.05553	109264.82	-0.17
		3, 4, 6, 11, 37			
10	2, 1,2, 3, 3, 3, 3, 1, 1, 2	16, 8, 4, 1, 1,	0.04167	109604.00	0.14
		4, 5, 7, 13, 8			
20	2, 1,2, 3, 3, 3, 3, 3, 1, 2	16, 8, 4, 1, 1,	0.04167	109765.86	0.29
		4, 5, 7, 13, 7			
30	2, 1,2, 3, 3, 3, 3, 1, 1, 2	16, 8, 4, 1, 1,	0.04167	109890.06	0.40
		4, 5, 6, 12, 7			

**Table 7:** When *S* changes, the optimal solution obtained by the IMFO algorithm.

***S***	**The optimal supplier mixture strategy**	**The optimal replenish strategy (*k***_i_*)	The optimal basic replenish cycle (T*)	The optimal total cost (TC*)
10	2, 1, 2, 3, 3, 3, 3, 1, 1, 2	12, 6, 3, 1, 1,	0.05553	109452.70
		3, 4, 5, 10, 6		
50	2, 1, 2, 3, 3, 3, 3, 1, 1, 1	8, 4, 2, 1, 1,	0.08330	110055.23
		2, 3, 4, 7, 25		
100	2, 1, 2, 3, 3, 3, 3, 1, 1, 3	8, 4, 2, 1, 1,	0.08330	110656.44
		2, 3, 4, 7, 25		
150	2, 1, 2, 3, 3, 3, 3, 3, 1, 1	6, 3, 2, 1, 1,	0.1132	111136.07
		2, 2, 1, 5, 18		
200	2, 1, 2, 3, 3, 3, 3, 1, 1, 2	4, 2, 1, 1, 1,	0.2000	111463.98
		1, 1, 2, 3, 2		
300	2, 1, 2, 3, 3, 3, 3, 1, 1, 2	4, 2, 1, 1, 1,	0.2000	111963.97
		1, 1, 2, 3, 2		
500	2, 1, 2, 3, 3, 3, 3, 1, 1, 2	4, 2, 1, 1, 1,	0.1997	112963.68
		1, 1, 2, 3, 2		
1000	2, 1, 2, 3, 3, 3, 3, 1, 1, 2	4, 2, 1, 1, 1,	0.2000	115463.97
		1, 1, 2, 3, 2		

Based on the computational results shown in Tables 5–7, the following observations can be derived:

1. The optimal total cost is weakly positively sensitive to changes of *h*_*i*_ and *S*, whereas the optimal total cost is highly positively sensitive to changes of *D*_*i*_. The demand rate has the strongest effect on the optimal total cost.
2. It can be found that the optimal solution (*k*_i_*, T*, *X*_ij_*) does not change regularly with the system parameters. The main reason lies in the fact that the proposed model considers the multi-supplier’s mixture strategy, where the different quantity discounts and the different minor ordering cost are obtained from different suppliers.
3. The optimal replenishment strategy (*k*_i_*) and the basic replenishment cycle (T*) are highly positively sensitive to the change of system parameters, while the optimal supplier-selection strategy remains relatively robust to the change. This indicates that the retailers may only need to redesign T* and *k*_i_*, and to keep the optimal solution of *X*_ij_* unchanged. This would save a great number of computational efforts while still achieving the optimal performance.

## 6. Conclusions

In this paper, we provide a pioneering focus on the JRP for multiple non-instantaneous deteriorating items with quantity discounts offered by multiple suppliers. A multi-supplier multi-item joint replenishment model is developed, aiming to minimize the average total cost. As this problem is NP-hard, a novel meta-heuristic algorithm, i.e., the MFO, is introduced and redesigned to solve the proposed model. From the results of numerical experiments, we can conclude that our proposed algorithm outperforms most well-known algorithms including GA, FOA, GWO, PSO, and MFO in both the solving efficiency and accuracy. Therefore, it could be considered as an alternative to solve optimization problems among the current algorithms. Sensitivity analyses of the key parameters indicate that the optimal replenishment policy and the basic replenishment cycle are highly positively sensitive to the changes of system parameters, while the supplier selection strategy is strongly robust to them.

This study provides a practical approach to daily inventory problems faced by managers who have to frequently replenish deteriorating items from multiple suppliers with quantity discounts. In real life, the coordinated replenishment with one-supplier offering different quantity discounts for different items could be regarded as a special case of the proposed model. To the best of our knowledge, our work is the first to investigate the joint replenishment problem for non-instantaneous deteriorating items with several suppliers offering different quantity discounts. Nevertheless, there are still some limitations in this study. For example, the demand and deterioration functions are assumed to be constant, and these assumptions may restrict the applications of the model. Moreover, the flame individual only records the historical optimal solution of its corresponding moth individual, and the update of the moth individual is always based on its corresponding flame individual. In other words, there is no communication among the moth individuals.

Our study provides several directions for future scholars in this research stream. For example, it would be interesting to investigate the optimal policy with more general demand rates. In addition, the promotional efforts and preservation technology investment for perishable products could be further considered. Furthermore, the algorithm can be extended to solve the JRP with resource constraints (such as carbon emission constraint, limited capital budget, etc.). In future research, once the better algorithms are developed, our IMFO algorithm could act as a valuable benchmarking algorithm.

## Supporting information

S1 Data The data set. (DOCX)
