# Supplementary material for: Optimizing multi-supplier multi-item joint replenishment problem for non-instantaneous deteriorating items with quantity discounts
Source: PLoS One. 2021 Feb 8;16(2):e0246035. doi: 10.1371/journal.pone.0246035 (PMC7870053; doi:10.1371/journal.pone.0246035)
Supplement: S1 Data — (DOCX) [file pone.0246035.s001.docx]

S1 Data. The Data Set.

**Table 2 Parameter settings of non-instantaneous deteriorating items (n=10)**

| **Item** | **1** | **2** | **3** | **4** | **5** | **6** | **7** | **8** | **9** | **10** |
| --- | --- | --- | --- | --- | --- | --- | --- | --- | --- | --- |
| ***s_i1_*** | 5.0 | 19.4 | 9.5 | 8.5 | 2.2 | 8.2 | 10.6 | 4.0 | 20.0 | 16.0 |
| ***s_i2_*** | 5.0 | 19.2 | 9.0 | 9.2 | 2.0 | 8.0 | 10.4 | 4.2 | 24.0 | 15.0 |
| ***s_i3_*** | 5.2 | 19.4 | 8.4 | 9.2 | 2.4 | 7.8 | 11.2 | 4.2 | 24.0 | 18.0 |
| ***D_i_*** | 600 | 900 | 2400 | 12000 | 18000 | 3000 | 2500 | 180 | 50 | 146 |
| ***h_i_*** | 0.5 | 1.94 | 0.95 | 0.85 | 0.22 | 0.82 | 1.06 | 0.4 | 2.0 | 1.6 |
| ***c_i_*** | 3 | 11.64 | 5.7 | 5.1 | 1.32 | 4.92 | 6.36 | 2.4 | 12 | 9.6 |
| ***θ_i_*** | 0.02 | 0.02 | 0.02 | 0.02 | 0.02 | 0.02 | 0.02 | 0.02 | 0.02 | 0.02 |
| ***t_di_*** | 0.0411 | 0.0822 | 0 | 0.0411 | 0.411 | 0.0822 | 0.0822 | 0 | 0.0411 | 0.822 |
| ***S*** | 10 | | | | | | | | | |

**Table 3 The quantity discount strategies provided by each supplier (* represent no price discount)**

| ***j*** | ***q_ijy_*** | ***C_1j_*** | ***C_2j_*** | ***C_3j_*** | ***C_4j_*** | ***C_5j_*** | ***C_6j_*** | ***C_7j_*** | ***C_8j_*** | ***C_9j_*** | ***C_10j_*** |
| --- | --- | --- | --- | --- | --- | --- | --- | --- | --- | --- | --- |
| 1 | *Q_i_*_1_<150 | 2.5 | 9.7 | 4.75 | 4.25* | 1.1 | 4.1 | 5.3 | 2.0* | 10.0* | 8.0 |
|  | 150≤*Q_i_*_1_<300 | 2.2 | 9.6 | 4.4 | - | 1.05 | 3.9 | 5.1 | - | - | 7.0 |
|  | *Q_i_*_1_≥300 | 2.1 | 9.4 | 4.1 | - | 0.95 | 3.7 | 4.8 | - | - | 6.0 |
| 2 | *Q_i_*_2_<200 | 2.5 | 9.6* | 4.5 | 4.6 | 1.0* | 4.0 | 5.2* | 2.1 | 12.0 | 7.5* |
|  | 200≤*Q_i_*_2_<400 | 2.2 | - | 4.2 | 4.25 | - | 3.8 | - | 2.05 | 8.0 | - |
|  | *Q_i_*_2_≥400 | 1.9 | - | 4.0 | 4.1 | - | 3.5 | - | 1.95 | 6.0 | - |
| 3 | *Q_i_*_3_<300 | 2.6 | 9.7 | 4.2* | 4.6 | 1.2 | 3.9 | 5.6 | 2.1 | 12.0 | 9.0 |
|  | 300≤*Q_i_*_3_<500 | 2.3 | 9.5 | - | 4.3 | 1.1 | 3.8 | 5.0 | 1.95 | 9.0 | 6.0 |
|  | *Q_i_*_3_≥500 | 2.0 | 9.3 | - | 4.0 | 0.9 | 3.4 | 4.6 | 1.90 | 5.0 | 5.0 |

**Notes:** parameters and data of our numerical experiments come from the following work:

Moon, I.K., Goyal, S.K. and Cha, B.C. The joint replenishment problem involving multiple suppliers offering quantity discounts. *International Journal of Systems Science*, 2008, 39(6): 629-637.
